# Supplementary material for: Incidence of Guillain-Barré Syndrome Is Not Associated with Influenza Vaccination in the Elderly
Source: Vaccines (Basel). 2020 Jul 31;8(3):431. doi: 10.3390/vaccines8030431 (PMC7563234; doi:10.3390/vaccines8030431)
Supplement: Supplementary file 1 [file vaccines-08-00431-s001.pdf]

Supplemental Materials

Supplemental Table 1. Precedent infections considered possible causes of Guillain-Barré syndrome

Precedent Infections	ICD-10th-codes
Upper respiratory	J00-J06, J20-J22, J40-42, H65-67
Lower respiratory	J440
Gastro-intestinal	A08-A09
Malaria	B50-B54, N080, P373, P374, T372, T378, Y412, Y418
Tsutsugamushi	A753
Other viruses	A92
Campylobacter	A045
Cytomegalovirus	B25
Epstein-Barr virus	B27
Herpes simplex virus	B00
Varicella (Chickenpox)	B01
Herpes zoster virus	B02
Mycoplasma	A493, B96, J29
Haemophilus influenzae	G00, J09-J18
Influenza virus	A413, A492, J201
Parainfluenza virus	J204, J2181
Other infections	B96, A045, P236

Supplemental Table 2. Results of conditional Poisson regression analysis by sex and age: subgroup analyses

Risk period (days)	Male (n = 168)			Female (n = 152)			65 – 74 years (n = 175)			75 – 84 years (n = 128)		
	IRR	95% CI		IRR	95% CI		IRR	95% CI		IRR	95% CI	
		Lower limit	Upper limit		Lower limit	Upper limit		Lower limit	Upper limit		Lower limit	Upper limit
Baseline (Before influenza vaccination or after vaccination of 90 days)	1.00	-	-	1.00	-	-	1.00	-	-	1.00	-	-
0-3	-	-	-	-	-	-	-	-	-	-	-	-
4-7	0.51	0.07	3.67	1.19	0.29	4.80	0.53	0.07	3.75	0.66	0.09	4.74
8-14	0.88	0.28	2.76	1.02	0.32	3.20	0.60	0.15	2.42	1.51	0.56	4.10
15-42	0.95	0.54	1.68	0.76	0.39	1.50	1.05	0.60	1.82	0.76	0.37	1.55
43-90	0.73	0.44	1.20	1.09	0.69	1.71	1.18	0.78	1.79	0.61	0.33	1.13

(CI, confidence interval; IRR, incidence rate ratio)
